# The unfolded protein response in neurodegenerative diseases: a neuropathological perspective

**DOI:** 10.1007/s00401-015-1462-8

**Published:** 2015-07-26

**Authors:** Wiep Scheper, Jeroen J. M. Hoozemans

**Affiliations:** Department of Clinical Genetics and Alzheimer Center, Neuroscience Campus Amsterdam, VU University Medical Center, Amsterdam, The Netherlands; Department of Functional Genome Analysis and Molecular and Cellular Neuroscience, Center for Neurogenomics and Cognitive Research, VU University, Amsterdam, The Netherlands; Department of Genome Analysis, Academic Medical Center, Amsterdam, The Netherlands; Department of Pathology, VU University Medical Center, PO Box 7057, 1007 MB Amsterdam, The Netherlands

**Keywords:** ER stress, Unfolded protein response, PERK, eIF2alpha, Neuropathology, Neurodegeneration

## Abstract

The unfolded protein response (UPR) is a stress response of the endoplasmic reticulum (ER) to a disturbance in protein folding. The so-called ER stress sensors PERK, IRE1 and ATF6 play a central role in the initiation and regulation of the UPR. The accumulation of misfolded and aggregated proteins is a common characteristic of neurodegenerative diseases. With the discovery of the basic machinery of the UPR, the idea was born that the UPR or part of its machinery could be involved in neurodegenerative diseases like Alzheimer’s disease, Parkinson’s disease, amyotrophic lateral sclerosis and prion disease. Over the last decade, the UPR has been addressed in an increasing number of studies on neurodegeneration. The involvement of the UPR has been investigated in human neuropathology across different neurological diseases, as well as in cell and mouse models for neurodegeneration. Studies using different disease models display discrepancies on the role and function of the UPR during neurodegeneration, which can often be attributed to differences in methodology. In this review, we will address the importance of investigation of human brain material for the interpretation of the role of the UPR in neurological diseases. We will discuss evidence for UPR activation in neurodegenerative diseases, and the methodology to study UPR activation and its connection to brain pathology will be addressed. More recently, the UPR is recognized as a target for drug therapy for treatment and prevention of neurodegeneration, by inhibiting the function of specific mediators of the UPR. Several preclinical studies have shown a proof-of-concept for this approach targeting the machinery of UPR, in particular the PERK pathway, in different models for neurodegeneration and have yielded paradoxical results. The promises held by these observations will need further support by clarification of the observed differences between disease models, as well as increased insight obtained from human neuropathology.

## The UPR, a highly conserved stress response

Neurodegenerative disorders like Alzheimer’s disease (AD), Parkinson’s disease (PD), prion disease, Huntington’s disease (HD), frontotemporal dementia (FTD), and amyotrophic lateral sclerosis (ALS) are characterized by the accumulation and aggregation of misfolded proteins. The proteins found in the aggregates and the brain areas where they accumulate are different for each neurodegenerative disease. Like all cells, neurons have an extensive system for protein quality control. This serves to detect and remove aberrant proteins, to prevent the detrimental aggregation process and deal with misfolding early in the process. A major site of protein synthesis is the endoplasmic reticulum (ER), where secretory, transmembrane and organelle-targeted proteins are synthesized, comprising approximately 30 % of the proteome. A key component of protein quality control in the ER is the unfolded protein response (UPR), which comes into play if the protein homeostasis (proteostasis) in the ER is disturbed.

Before the UPR was discovered, it had already been observed that different types of cellular stress like viral transformation, inhibition of glycosylation and calcium ionophore treatment induced the expression of a select group of proteins. These proteins were called glucose-regulated proteins (GRPs) because of their induction by glucose deprivation and to distinguish them from a related group of proteins that were induced by heat, the heat-shock proteins [53, 104]. In 1988, the first direct connection between protein folding stress in the ER and the induction of GRPs, including GRP78 (BiP), was made by overexpression of mutant influenza hemagglutinin protein in mammalian cells [52]. This stress response was thus designated unfolded protein response or UPR. Gradually, the key signaling events that mediate the response were identified, with pioneering work done in yeast, demonstrating that a specific promoter element is responsible for the transcriptional upregulation of GRPs and other targets [67]. This was followed by the identification of the sensor in the ER membrane responsible for transducing the signal from the misfolded proteins in the ER to the nucleus (Ire1p/Ern1p), reported more or less simultaneously by two groups [18, 66]. Two mammalian homologues (IRE1α and β) were identified a few years later [109, 119]. Ire1p oligomerizes when the response is triggered which results in trans-autophosphorylation [94, 120]. An important result of activation of Ire1p is the unconventional splicing of Hac1p mRNA, resulting in the generation of the active transcription factor Hac1p [19, 98]. The mammalian substrate of the IRE1 endonuclease, XBP1 mRNA, has remained elusive for a long time as it bears no homology to Hac1p. Nonetheless, the mechanism of activation by unconventional splicing is conserved [11, 55, 126].

The yeast UPR is mediated entirely by the Ire1p pathway, but metazoans have additional sensors and, as a result, more downstream targets and broader cellular effects. It was observed that during activation of the UPR in mammalian cells protein synthesis is inhibited by phosphorylation of the translation initiation factor eIF2α, as is also a common response to other types of cellular stress [later termed the integrated stress response (ISR), see below]. However, none of the eIF2α kinases known at the time were activated by ER stress. Protein kinase R (PKR)-like endoplasmic reticulum kinase **(**PERK), an ER transmembrane protein, was later identified as this novel eIF2α kinase [33, 95]. It combines the interesting properties of a luminal domain highly homologous to IRE1 to sense misfolded proteins in the ER connected to a cytosolic kinase domain that resembles the other eIF2α kinases. Mammalian cells contain another ER stress transducer, the third in line to be discovered, activating transcription factor 6 (ATF6). This membrane-bound transcription factor is transported to the Golgi upon UPR activation where it is processed and released to the nucleus [34, 125]. As for IRE1, for ATF6 also two isoforms exist, ATF6α and ATF6β.

**Fig. 1:**
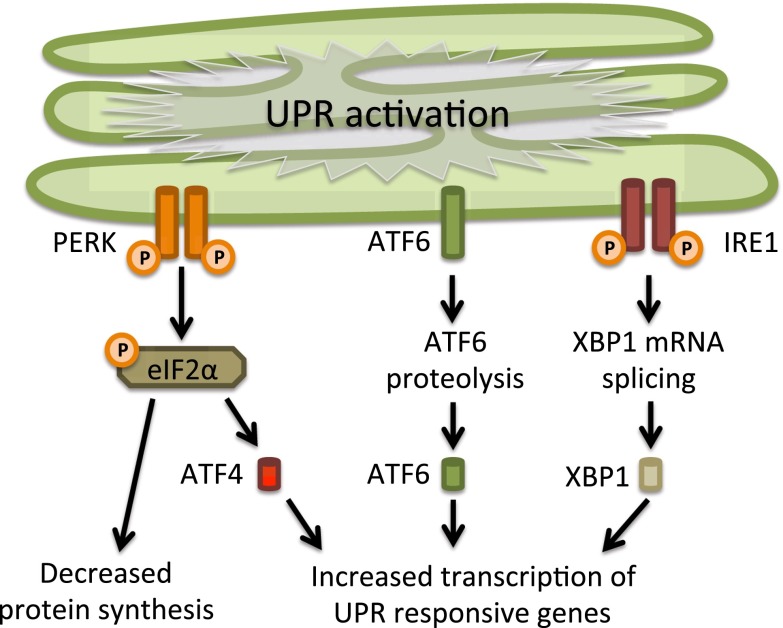
The unfolded protein response. The unfolded protein response consists of three independent signaling pathways that work in parallel and are activated upon accumulation of unfolded proteins inside the ER. Each signaling pathway is defined by the different ER-resident transmembrane proteins that act as ER stress sensors: RNA-activated protein kinase R (PKR)-like ER kinase (PERK), activating transcription factor 6 (ATF6) and inositol requiring enzyme 1 (IRE1). Activation of the UPR leads to an overall translational block and specific activation of ER stress responsive genes, which will increase the protein folding capacity and decrease the protein folding load in the ER. See text for further details

The IRE1, PERK and ATF6 pathways together comprise an intricate network that has a broad range of transcriptional and translational targets. The UPR is closely connected to the proteolytic machinery of the cell. Proteins that misfold in the ER are exported to the cytosol and degraded by the proteasome [80]. However, once the UPR is activated, autophagy is increased and this becomes the major proteolytic system [5, 24, 69, 73, 90]. Although many mechanistic details and additional regulatory pathways are still being uncovered, the core signaling of the mammalian UPR had been unraveled by 2002 (Fig. 1).

An important function of the UPR is its function as a homeostatic stress response initiated by ER dysfunction. In addition, it is employed to adjust the physiology of cells under situations where ER function is not impaired [81]. For example, during the differentiation of B-cells to antibody-producing plasma cells, pathways of the UPR are employed to expand the ER [46]. Also in cells that demonstrate a highly dynamic physiologically regulated range of secretory demand, like insulin secretion in pancreatic β-cells, the UPR is involved [91]. Not surprisingly, dysfunction of the UPR can therefore give rise to disease. For example, Wolcott–Rallison syndrome is a rare hereditary disease caused by loss of function of PERK [23]. Individuals with this disease develop defects that are connected to loss of the physiological function of the UPR, including diabetes due to loss of β-cell function. A completely opposite way in which the UPR can lead to disease is observed in cancer, where hyperactivity of the UPR facilitates the survival of tumor cells [63]. In contrast, in neurodegenerative diseases—the focus of this review—UPR activation is connected to ER dysfunction and leads to loss of neuronal function. It is important to be aware of the different faces that the UPR has in physiology and pathology.

Because accumulation of misfolded proteins is a common characteristic of neurodegenerative diseases, it is not surprising that the involvement of the UPR during neurodegeneration has been extensively studied in both in vitro and in vivo models (for review see [79]). It is becoming apparent that the role of the UPR in these models is not always consistent and sometimes even paradoxical (see detailed discussion below). The proposed functional role of the UPR concluded from these models is often difficult to connect to the situation in the human brain under pathological conditions. To understand the impact or relevance of the UPR in vitro or in vivo using models for neurodegeneration a direct relation should be made with human neuropathology. In 2005, our group reported activation of the UPR in human AD brain [42]. The investigation of many different neurodegenerative diseases in even more different model systems has increased enormously. Below, we will discuss the progress made in UPR research in neurodegenerative diseases over the last decade. We will specifically address similarities and discrepancies between observations in human pathology and disease models.

## UPR activation in human neuropathology

To detect UPR activation in samples of human brain, different methods can be employed. Altered expression of UPR target genes can be determined by analysis of mRNA expression in brain lysates. This is a sensitive method, but has the disadvantage that changes in only a subset of the cells may not be detected because they are diluted out. The same limitation applies to measuring protein levels of UPR markers in protein lysates. Currently, many antibodies are available directed to the main players of the UPR that enable studying its activation using different techniques. It should be noted that determination of UPR protein levels not always allows assessment of UPR activation since part of the UPR relies on mechanisms that involve protein cleavage, post-translational modification, intracellular distribution or altered conformation.

For detection of UPR activation, phospho-specific antibodies that specifically detect the active, phosphorylated, forms of the ER stress sensors PERK and IRE1 have become an important tool. Also, for the phosphorylated substrate of PERK, p-eIF2α, phospho-specific antibodies are available, but this is not a specific UPR marker, because it is the converging point of the ISR. The ISR involves apart from PERK three other stress-induced eIF2α kinases, PKR (protein kinase double-stranded RNA-dependent), GCN2 (general control non-depressible-2), and HRI (heme-regulated inhibitor) [25]. In addition, immunohistochemistry or immunofluorescence can be employed for UPR-related translocation events, of the transcription factors ATF6 and XBP1 to the nucleus. In addition, ATF4 and CHOP positive nuclei are in accordance with UPR activation, but again these downstream targets in the PERK pathway are not specific UPR markers because of the presence other eIF2α kinases. An additional advantage of UPR detection in situ by immunohistochemistry or immunofluorescence is that it can be pinpointed to specific cells (e.g., neurons or glia) and directly correlated to pathological hallmarks. Using above-described methods, different UPR markers have been observed in different neuropathological conditions (Table 1).

**Table 1:** UPR markers in human neuropathology

Neurodegenerative disease		UPR marker	Technique, brain area	Association with pathology	References
Alzheimer’s disease		GRP78	IHC, hippocampus	Increased in AD, associated with healthy neurons	[29]
		p-eIF2α	IHC, hippocampus, entorhinal cortex	Increased in AD, associated with GVD	[15]
		pPERK, GRP78	IHC and WB, hippocampus and temporal cortex	Increased levels in AD	[42]
		pPERK, p-eIF2α	IHC, hippocampus, frontal cortex	Increased in AD, associated with abnormally phosphorylated tau	[111]
		hHRD1	IHC, hippocampus	Increased in AD	[43]
		pPERK, pIRE1, p-eIF2α	IHC, hippocampus	Increased in AD, associated with GVD and abnormally phosphorylated tau	[41]
		pPERK, p-eIF2α	IHC, pons medulla, hippocampus	Increased in affected brain areas	[103]
Tauopathy					
CBD/PSP		pPERK, p-eIF2α	IHC, hippocampus, frontal cortex	Increased in CBD/PSP, associated with abnormally phosphorylated tau	[111]
FTDP-17T, PiD, PSP		pPERK, pIRE1	IHC, hippocampus, frontal cortex, temporal cortex	Increased in affected brain areas, associated with GVD and early tau pathology	[70]
PSP		pPERK, p-eIF2α	IHC, pons medulla, hippocampus	Increased in affected brain areas	[103]
Synucleinopathy					
Parkinson’s disease		pPERK, p-eIF2α	IHC, substantia nigra	Increased in PD, association with α-synuclein	[40]
Multiple system atrophy		pPERK, pIRE1, p-eIF2α	IHC, middle cerebellar peduncle, white matter of cerebellum, pontocerebellar fibers, striatum, GCI	Association with α-synuclein inclusions, abnormally phosphorylated tau, pTDP-43 and GVD	[61]
Prion disease (sCJD, vCJD)		GRP58, GRP78, GRP94	WB, cortex	Increased levels in CJD	[36]
		pPERK, p-eIF2α	IHC, hippocampus, frontal cortex	No increase in CJD	[111]
Amyotrophic lateral sclerosis		PERK, ATF6, IRE1, GRP78, Erp57, PDI, CHOP, caspase 4	WB, spinal cord	PERK, ATF6, IRE1 and caspase 4 are in increased in ALS. GRP78, Erp57, PDI and CHOP are unchanged	[2]
		CHOP	IHC, spinal cord	CHOP is increased in ALS	[45]
		GRP78	IHC, spinal cord	GRP78 is increased in ALS	[86]
		p-eIF2α	IHC, WB, spinal cord	p-eIF2α is increased in ALS	[44]
		XBP-1s, ATF4, GRP58	WB, spinal cord	XBP-1s, ATF4 and GRP58 are increased in ALS	[37]
Repeat expansion diseases					
Huntington’s disease		GRP78, CHOP	PCR, parietal cortex	Increased expression in HD	[12]
		ATF6α	IHC, WB, caudate putamen	Impaired ATF6α processing	[26]
		pIRE1, GRP78	WB, striatum	Increased levels in HD	[54]
		XBP-1s, ATF4, CHOP, GRP78	WB, striatum	Increased levels of XBP-1s, no changes in ATF4, CHOP, GRP78	[114]
C9ALS		ATF4, CHOP, GRP78	PCR, frontal cortex	Increased levels of ATF4 and CHOP in C9ALS, no changes in GRP78	[130]

*UPR* unfolded protein response, *GRP* glucose-regulated protein, *IHC* immunohistochemistry, *AD* Alzheimer’s disease, *p-eIF2α* phosphorylated eukaryotic initiation factor 2 alpha, *GVD* granulovacuolar degeneration, *pPERK* phosphorylated protein kinase R (PKR)-like endoplasmic reticulum kinase, *WB* Western blot analysis, *hHRD1* ERAD-associated E3 ubiquitin-protein ligase, *pIRE1* phosphorylated inositol requiring enzyme 1, *CBD* corticobasal degeneration, *PSP* progressive supranuclear palsy, *FTDP-17T* hereditary FTD and parkinsonism linked to chromosome 17, *GCI* gyrus cinguli, *s/vCJD* sporadic/variant Creutzfeldt–Jakob disease, *ATF* activating transcription factor, *PDI* protein disulfide isomerase, *CHOP* C/EBP homologous protein, *XBP-1s* X-box binding protein 1s isoform, *ALS* amyotrophic lateral sclerosis, *PCR* polymerase chain reaction, *HD* Huntington’s disease, *C9ALS* ALS with the C9ORF72 repeat expansion

### Alzheimer’s disease

**Fig. 2:**
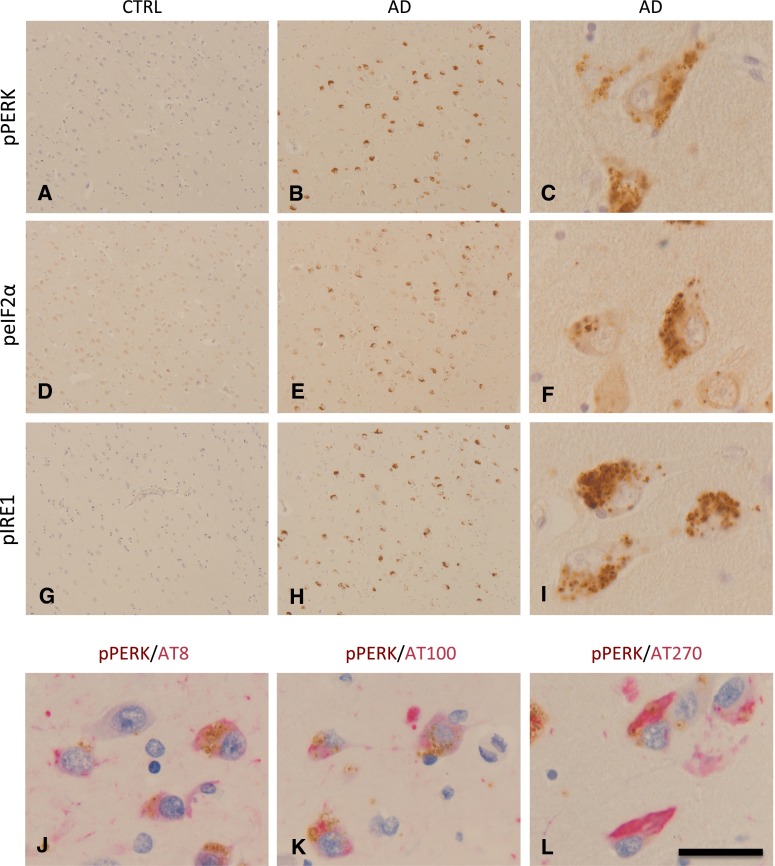
UPR activation in Alzheimer’s disease. Immunohistochemical detection and antibodies used for the detection of UPR markers and phosphorylated tau (AT8, AT100 and AT270) have been described previously [41, 42]. Shown are pictures of the hippocampal sub-area subiculum of a control case (CTRL, Braak 0) and an AD case (Braak 5). **a**–**c** pPERK is detected by immunohistochemistry in pyramidal neurons of an AD case and is absent in a control case showing no AD pathology. pPERK is present in granules which can be defined as granulovacuolar degeneration. **d**–**f** p-eIF2α immunohistochemistry on the same area shown for the control and AD case in **a**–**c**. Also p-eIF2α can be detected as granules in pyramidal neurons. **g**–**i** pIRE1α is also detected in pyramidal neurons in the subiculum of an AD case and is absent in a control case (shown is the same area as indicated in **a**–**c**). Similar granular structures are detected as observed with pPERK and p-eIF2α immunohistochemistry. **j**–**k** UPR markers in AD are localized in neurons showing increased presence of phosphorylated Tau protein;** j** Double immunolabeling for pPERK (*brown*) and AT8 (*red*, pTau Ser202), **k** pPERK (*brown*) and AT100 (*red*, pTau Ser212 and Thr 214) and **l** pPERK (*brown*) and AT270 (*red*, pTau Thr181). Sections were counterstained with haematoxylin (*blue*). *Scale bar* **a**, **b**, **d**, **e**, **g**, **h** 300 μm; **c**, **f**, i–**l** 40 μm

Alzheimer’s disease (AD) is the most prevalent neurodegenerative disease and the most common form of dementia. Deposits of aggregated proteins are a prominent neuropathological hallmark of AD: intracellular aggregates of tau in the neurofibrillary tangles (NFTs), dystrophic neurites and neuropil threads, and extracellular aggregates of β-amyloid (Aβ) in the senile plaques. AD thus represents a prime example of a protein folding disease [106]. Markers specific for UPR activation are increased in AD brain tissue compared to non-demented control brain tissue (Fig. 2). GRP78 is increased in AD in the hippocampus and temporal cortex and various studies from different groups have shown increased presence of phosphorylated (p)PERK, pIRE1, and p-eIF2α in AD neurons [15, 29, 41, 42, 103, 111]. These markers appear either in morphologically healthy neurons or in neurons with abnormally phosphorylated tau protein, but are almost absent from NFT-containing neurons. Overall, the levels of GRP78 and the occurrence of pPERK in AD neurons correlate very well with the presence of abnormally phosphorylated tau and the Braak staging for NFTs [41]. These observations indicate that the UPR is involved in the early stages of AD pathology.

### Non-AD tauopathies

Neurodegenerative diseases that show a primary pathology consisting of inclusions of filamentous tau can be designated as a tauopathy and include diseases like sporadic corticobasal degeneration (CBD), progressive supranuclear palsy (PSP), Pick’s disease (PiD), as well as hereditary FTD and parkinsonism linked to chromosome 17 (FTDP-17T). Different groups have now shown increased presence of pPERK, p-eIF2α and pIRE1 in affected brain areas in these tauopathies [70, 103, 111]. From these studies, it appears that UPR activation markers occur in cells, i.e., neurons and glia that show abnormal tau phosphorylation, suggesting that UPR activation and tau phosphorylation are closely linked during neurodegeneration.

### Synucleinopathies

Parkinson’s disease (PD) is characterized by the selective loss of dopaminergic neurons in the substantia nigra pars compacta (SN) and the accumulation of α-synuclein in Lewy bodies. The involvement of the UPR in PD has primarily been shown in in vitro models [82]. Although a role for the UPR in neuronal cell death in PD pathogenesis is widely suggested, there is hardly any data on UPR activation from postmortem studies on PD cases. Our group investigated the immunohistochemical localization of pPERK and p-eIF2α in the substantia nigra of PD and control cases [40]. Immunoreactivity for pPERK and p-eIF2α is observed in PD in neuromelanin containing neurons of the SN, while these markers are absent in control cases. Multiple system atrophy (MSA) is a sporadic neurodegenerative disease that is also characterized by intracellular accumulation of α-synuclein. In MSA, pPERK, p-eIF2α, and pIRE1 were increased in and closely associated with glial cytoplasmic inclusions containing α-synuclein during the initial state of deposition [61]. The close association between UPR markers and accumulation of α-synuclein in the cytoplasm suggests a strong relation between α-synuclein and ER stress. This is supported by in vitro models showing that overexpression of wild-type or mutant α-synuclein increases the vulnerability for ER stress through various mechanisms [17, 100].

### Prion disease

Prion disease or transmissible spongiform encephalopathies (TSEs) are fatal neurodegenerative disorders (e.g., Creutzfeldt–Jakob (CJD), Gerstmann–Sträussler–Schenker disease (GSS), fatal familial insomnia (FFI), and Kuru), which are characterized by rapidly progressing neuronal loss and extracellular accumulation of the scrapie form of the prion protein (PrP^Sc^), a pathological isoform of the normal cellular prion protein (PrP). Detection of UPR activation markers, especially phosphorylated proteins, could be difficult in human prion disease due to the relatively long postmortem delay as a result of infectivity precautions. In 2003, Hetz and colleagues reported on increased caspase-12 activation and elevated levels of ER stress markers GRP58, GRP78 and GRP94 in cortical samples from sporadic CJD and variant CJD cases [36]. The role of caspase-12 in neurodegeneration in general and in human neuropathology in particular is debatable. In mice, caspase-12 is also involved in the innate immune responses by regulating the processing of inflammatory cytokines and caspase-12 deficiency in mice confers resistance to sepsis [83]. In the great majority of the human population, however, caspase-12 is expressed as a truncated, catalytically inactive protein. A subset of individuals of African descent expresses full-length caspase-12 rendering these individuals more susceptible to inflammatory diseases, again pointing more to a key role for this caspase in the immune response [84]. These issues should be kept in mind considering the interpretation of caspase-12 activation in human brain tissue samples.

An extensive immunohistochemical study looking at the localization of pPERK and p-eIF2α could not confirm the involvement of the UPR in CJD [111]. Only CJD cases that showed concomitant AD pathology had increased presence of pPERK and p-eIF2α, suggesting that these markers were not related to the prion pathology. This indicates that comorbidity or co-occurrence of neuropathological processes is an important factor in the study of UPR activation in human neuropathology. Definite conclusions can only be made when brain tissue is neuropathologically assessed for different pathological hallmarks, particularly abnormally phosphorylated tau. Whether other arms of the UPR than the PERK pathway are involved in human CJD pathology needs to be addressed in future studies.

### Amyotrophic lateral sclerosis

Amyotrophic lateral sclerosis is characterized by the degeneration of motor neurons in the spinal cord, cortex and brain stem, leading to muscle atrophy and paralysis [10]. Protein levels of total PERK, ATF6, IRE1 and caspase-4 are increased in the spinal cord of sporadic ALS patients [2]. In addition, increased levels of XBP-1s, ATF4 and GRP58 have been observed in human postmortem spinal cord samples of sporadic ALS patients by Western blot analysis [37]. Immunohistochemical analyses indicate an increase in CHOP and GRP78 in ALS spinal cord [45, 86]. pPERK and p-eIF2α have been observed in the spinal cord of transgenic mice models for ALS [68]. To our knowledge, there are no reports on increased levels of pPERK in ALS spinal cord. By both immunohistochemistry and Western blot analyses, increased levels of p-eIF2α are detected in spinal cord samples from patients with sporadic ALS compared control cases [44]. UPR activation has been thoroughly investigated in models for ALS and increased levels of a variety of UPR markers have been reported in spinal cord samples from ALS patients. However, it should be noted that comparative studies on human postmortem spinal cord samples from ALS patients and matched control cases to date only comprised low number of cases making statistical analysis difficult.

### Repeat expansion diseases

Expanded polyglutamine (polyQ) repeats found in different proteins can cause human-inherited neurodegenerative diseases, such as Huntington’s disease (HD), spinobulbar muscular atrophy, dentatorubal-pallidoluysian atrophy and spinocerebellar ataxia (SCA). These disorders are characterized by accumulation of intracellular protein aggregates and selective neuronal death. Expression levels of GRP78 and CHOP mRNA were found to be increased in the parietal cortex of HD patients compared to control cases [12]. Increased protein levels of pIRE1 and GRP78 can be observed in striatal tissue of HD patients compared with controls by Western blot analysis [54]. Vidal and colleagues have reported increased protein expression of XBP-1s in the striatum of a subset of HD cases compared with control cases, while no detectable changes in protein levels were observed for ATF4, CHOP, and GRP78 [114]. Another study showed that the processing of ATF6 to its active nuclear form is impaired in affected brain regions of Huntington’s disease patients [26].

A G_4_C_2_·G_2_C_4_ repeat expansion in a non-coding region of the *C9ORF72* gene is the most common genetic cause of ALS and FTLD-TDP [22, 78]. In the frontal cortex, mRNA levels of ATF4 and CHOP are significantly increased in ALS patients with the C9ORF72 repeat expansion compared to ALS patients without the repeat expansion, whereas no differences in GRP78 mRNA levels were observed [130].

From observations in postmortem brain, it is hard to draw conclusions about the involvement of the UPR in repeat expansion diseases. Most studies have been performed with a low number of disease and control cases and do not show the association with the extent of pathology or the number of repeat expansions in the affected genes. This makes statistical analysis and interpretation of data very difficult. More extensive studies on UPR markers are required to determine the role of the UPR in repeat expansion diseases.

### UPR markers are associated with granulovacuolar degeneration

In various neurodegenerative diseases (AD, tauopathies, MSA), UPR activation markers are observed in neuropathological structures that are defined as granulovacuolar degeneration (GVD). GVD is characterized by basophilic granules surrounded by a clear zone measuring 1–5 μm in diameter, occurring predominantly in hippocampal neurons [74, 107]. It is reported that GVD occurs in adult control brains and increases slightly with increasing age, however, the occurrence of GVD in AD brain is increased compared to age-matched control brain [4, 122]. In addition, the occurrence of GVD is associated with pathological hallmarks and clinical signs of AD as it correlates with the presence of NFTs, neuritic plaque pathology, Aβ-protein deposition phases, cerebral amyloid angiopathy stages and clinical dementia rating (CDR) scores [107]. Currently, the molecular events in GVD-containing neurons are poorly understood. Histochemical and ultrastructural observations suggest that GVD may correspond to a special type of autophagosome [74]. The presence of UPR activation markers in GVD granules may be explained by inclusion of ER-derived material in the autophagosomes. Alternatively, there is evidence indicating that the ER can serve as a membrane source for autophagosome formation [6].

### Conclusions from neuropathogical studies

All neurodegenerative diseases described above show features of an activated UPR. The most elaborate insight with regard to the association with pathological hallmarks and disease progression has been obtained for AD. Insight in the involvement of the UPR in different pathological stages (e.g., Braak stage for NFT of LB, Thal staging for amyloid β [8, 9, 108]) will provide directions for functional studies into the involvement of the UPR in neurodegenerative models, and feasibility of potential therapeutic approaches. For most neurodegenerative diseases studied, the assessment of the three arms of the UPR together is lacking, although this is important in view of crosstalk and compensation between the pathways (see detailed discussion below). Several factors can directly or indirectly influence the activity of the three ER stress transducers which may fine-tune the output of the UPR in physiological conditions. This has been best studied for the IRE1 pathway (reviewed in [35]). IRE1 has been implicated in determining the switch from adaptive to apoptotic signaling if the stress is prolonged, which is then followed by apoptosis [30]. However, prolonged activity of all 3 branches is observed in several neurodegenerative diseases in the absence of signs of apoptosis. This indicates that the dysregulated UPR activity in pathological situations is very different from that observed in physiological cell models. A noteworthy observation across the different neurodegenerative diseases is the association of UPR activation markers with the occurrence of early signs of tau pathology. In AD, CBD, PSP, PiD, FTDP-17T and MSA, UPR activation is found in neurons that show accumulation of abnormally phosphorylated tau. These observations across different diseases strengthen the hypothesis that UPR activation and abnormal tau phosphorylation/aggregation are functionally connected.

## UPR activity in models for neurodegenerative disease: truth or artifact?

To model neurodegenerative diseases, overexpression of aggregating proteins and more often of mutant derivatives associated with familial variants of the disease is used. Typically, this models only part of the pathogenesis in an exaggerated and accelerated fashion. This is useful for some purposes, but also creates an artifact-prone situation, in particular for a response that is designed to detect protein stress. A good example of ambiguous results is Presenilin 1 (PS1), mutations in which are the most common cause of autosomal dominant inherited forms of AD. PS1 was reported to affect the signaling of the UPR in models using overexpression [48] as well as knockout [71]. In contrast, other labs did not observe effects of PS1 mutant overexpression or deficiency on the UPR [75, 87, 101]. The exact cause of these differences is not known, and may relate to different cells, promoters and expression levels, mouse lines, specific mutations in PS1 used, etc. In any case, it is clear that disturbed UPR signaling is not a common feature of PS1 mutations and, in addition, it is elusive whether UPR signaling is activated and involved in the pathogenesis of AD in PS1 mutation carriers.

Several groups reported that exogenous application of synthetic Aβ induces or potentiates the UPR, albeit to different extent [14, 105, 127]. What is important to realize is that the local amounts of aggregates in these experimental setups exceeds that observed in the brain excessively. In an animal model for prion disease, injection of PrP^sc^ in the brain of mice results in UPR activation [65]. Although the exposure to PrP^sc^ reflects the pathogenesis of the human sporadic disease relatively well, in most experiments it still involves exposure to higher levels of aberrant proteins in Tg mice that express higher levels of the normal PrP^c^ to speed up the pathology. The flooding of neurons and synapses with toxic aggregates may lead to a disturbance in the ER, however, this may relate to a more general disturbance of cell physiology rather than a specific effect on the UPR. It was reported that UPR activation is also observed in the absence of overexpressed PrP^c^ [65] and although this will increase the time for phenotypes to develop this may be a more artifact-free model for the human disease.

This indicates another important issue: The aggregating proteins in neurodegenerative disease do typically not accumulate in the ER and many of them do not enter the ER at any stage in their life cycle. Effects on UPR signaling may therefore be indirect or not even directly related to ER stress. For example in the prion disease model, PERK activation does not seem to be accompanied by activation of the other two UPR branches, which makes it a very specific type of PERK activation, possibly not via ER stress [65, 72]. In overexpression models for α-synuclein [17], it was shown that accumulation of α-synuclein in the cytosol blocks ER–Golgi trafficking, leading to reduced ER exit and induction to the UPR. In another example, our own lab found that incubation of neuronal cells in culture with neurotoxic concentrations of Aβ oligomers did not induce a robust UPR within 48 h [14], although the uptake of oligomers occurs within minutes after application [13]. The oligomers did not directly encounter the ER, but did sensitize cells for a secondary ER stress insult. It is for example possible that the oligomers disturb intracellular calcium homeostasis via their toxic effect on mitochondria and lysosomes, thus indirectly affecting calcium homeostasis in the ER.

The lack of a direct colocalization between the disease causing proteins and the ER has prompted research into investigating the connection the other way around and consider the option that UPR activation precedes and facilitates pathology. There is for example no evidence for UPR activation by Aβ pathology in APP/PS1 mice. These only show UPR activation in aged mice, despite extensive pathology much earlier in younger mice [50].

Recent studies show that in animal models for prion disease and Aβ pathology increased PERK activity results in chronic inhibition of protein synthesis by eIF2α phosphorylation [60, 64, 65]. This prolonged UPR activation results in reduced levels of synaptic proteins and induces synaptic loss and neurodegeneration. This is an exciting new view on how chronic activation of the UPR facilitates neurodegeneration [89]. Although overall translation is inhibited by eIF2α phosphorylation, the translation of a select set of mRNAs is increased under these conditions. The mRNA encoding BACE1, a key enzyme in Aβ formation, was demonstrated to be one of these transcripts. BACE1 is thus subject to PERK-mediated translational upregulation via eIF2α phosphorylation. This UPR-induced increase in BACE1 levels results in enhanced Aβ production in Tg2576 mice [72]. This corroborates with an earlier report showing that UPR activation increases the formation of Aβ in PS1 mutant fibroblasts [75] although in this study the involvement of the PERK pathway was not specifically addressed.

As was observed in the APP/PS1 mice also in transgenic tau mice (P301L), the UPR is activated only in aged mice [38, 50]. UPR activity occurs therefore well after the occurrence of tau pathology, which makes it unlikely that pathological tau induces the UPR. In contrast, both in cell culture [113] and animals [56] endogenous tau is phosphorylated at disease relevant epitopes upon induction of the UPR. This suggests that activation of the UPR facilitates tau pathology. Results from our lab indicate that initially the UPR-induced tau phosphorylation is reversible and may be part of the adaptive response to stress [113]. However, prolonged UPR activation and tau phosphorylation as occurs in the brains of tauopathy patients may facilitate the formation of irreversible tau aggregates. In a very aggressively progressing tau mouse model (Tg4510) that shows extremely rapid tau aggregation and neuronal loss, it was shown that the tau aggregates impair ER proteostasis, thus contributing to activation of the UPR [1]. This may in turn result in a vicious cycle once aggregates form and may explain the UPR induction in aged tau mice [102]. The UPR-induced tau phosphorylation can be inhibited using a small molecule inhibitor of the PERK pathway [113], suggesting the involvement of this pathway.

Interestingly, subtle changes in UPR activity could bear relevance in human disease. Recently, this has gained further support from genetic studies that associate the *EIF2AK3* gene with increased risk of the tauopathies PSP and AD [39, 58]. The *EIF2AK3* risk allele was shown to increase the signaling activity of the PERK pathway [57]. Likewise, a polymorphism in the *XBP1* gene, which encodes the transcription factor activated by the IRE1 branch of the UPR, was identified as a genetic risk factor for AD [59]. The polymorphism affects the expression of XBP-1 and thereby the signaling activity in the IRE1 pathway [47, 77].

Despite the use of different animal and cell models for some specific mechanistic questions, many of these are quite different from the human disease. Recent advances in induced pluripotent stem cell (iPSC) technology lead the way to the generation of disease relevant human neurons. Cortical neurons derived from sporadic AD and APPE693Δ fAD fibroblasts showed extensive intracellular Aβ oligomer accumulation and increased GRP78 mRNA levels in particular in the fAD mutant cells, but involvement of other components of the UPR was not reported [51]. Human motor neurons derived from SOD1 A4V fALS mutation carrier fibroblasts causes hyperexcitation associated with upregulation of XBP-1s and increased p-eIF2α [115]. Inhibition of the hyperexcitation reduces the levels of XBP-1s, indicating that it is downstream of the electrophysiological phenotype [115]. It was suggested that this could induce a vicious cycle, because UPR induction has been shown to increase activity in motor neurons [49]. The exact mechanism needs further investigation, because if the signaling via the PERK pathway was prolonged using Salubrinal treatment the neuronal activity was actually reduced. Interestingly, these events all preceded the aggregation of the mutant SOD protein [49]. The data suggested that the levels of UPR target proteins are relatively high in wild-type motor neurons, indicative of basal UPR activation. Because this is associated with a relatively high sensitivity to ER stress, this could be an interesting explanation for the selective motor neuron pathology in ALS. The developments in the technology to culture human neurons create an elegant model to further elaborate on this, also in less-severe disease variants than the A4V mutant [92]. Cortical neurons were derived from A53T α-synuclein fibroblasts to establish a model for cortical synucleinopathy [16]. As was shown in yeast and mammalian cell models before, these cells display accumulation of ERAD substrates in the ER and increased levels of the UPR targets GRP78 and PDI. Also, in this case, the added value of human neurons was indicated, as all these phenotypic changes required neuronal differentiation.

## Targeting the UPR

Many models for neurodegenerative disease show UPR activity, but how do changes in UPR signaling affect the neurodegenerative process? This is important from mechanistic point of view, but also when considering targeting of the UPR for treatment of neurodegenerative disease.

Knockout mice for the UPR sensors were generated already early after their discovery and very severely affect the development and physiology of the animals. Homozygous PERK−/− mice have a phenotype very similar to humans with Wolcott–Rallison syndrome, in which the gene encoding PERK (*EIF2AK3*) is mutated [23]. Very pronounced is the defect in the function of the endocrine and exocrine pancreas, resulting in many systemic problems and early mortality [31, 129]. PERK-deficient cells are more sensitive for ER stress [32]. ATF6α deficiency also increases sensitivity for ER stress and *ATF6αβ* double knockouts are embryonic lethal [121, 123, 124]. Also, germline deletion of *XBP1* [76] or *IRE1α* [128] in mice is embryonic lethal.

The apparently increased sensitivity for ER stress in carriers of the UPR risk alleles may result in pathology in the long run. More research will be needed to establish how these risk variants contribute to pathology. The existence of risk variants may imply that also protective variants exist. In addition, if subtle increases in UPR signaling activity enhance risk, this could mean that subtle inhibition of activity by pharmacological intervention may be a viable approach. PERK and IRE1 are considered to be “druggable” and the list of small molecule inhibitors to target these UPR sensors is growing [62].

For IRE1, both RNase and kinase inhibitors have been developed that differentially affect the respective activities and the dimerization properties of IRE1. Advantage of just inhibiting the RNase may be that only the XBP-1 processing is inhibited, whereas phosphorylation of putative other substrates of the IRE1 kinase and its dimerization are not affected [20, 85]. Type I kinase inhibitors inhibit autophosphorylation, but stimulate RNase activity, which may be useful for research, but not for clinical development [117]. Type II inhibitors inhibit both kinase and RNase activities and thus effectively block all signaling via IRE1 [27]. In models for ER stress-mediated degeneration the type II IRE1 inhibitor KIRA6 promotes cell survival [27].

Targeting of the PERK/eIF2α pathway has received a lot of attention the last couple of years (Table 2). An early breakthrough was the compound Salubrinal, which targets the regulatory subunits of the eIF2α protein phosphatase 1c (PP1c) [7]. Salubrinal was shown to ameliorate the neurodegenerative phenotype in a mouse model for ALS [88]. The drug Guanabenz, which is an α_2_-adrenergic receptor agonist used to treat hypertension, was demonstrated to selectively inhibit the stress-induced eIF2α protein phosphatase regulatory subunit 15 A (PPP1R15A; a.k.a. GADD34, growth arrest and DNA damage-inducible protein 34) that forms a complex with PP1c [110], whereas Salubrinal also targets the constitutive PPP1R15B-PP1c complex. Guanabenz therefore does not completely inhibit the dephosphorylation of eIF2α. Guanabenz was beneficial in a SOD1 as well as a TDP-43 transgenic mouse model [112, 118].

**Table 2:** Small molecules targeting the PERK pathway of the UPR: effects in mouse models for neurodegenerative disease

Compound	Target	p-eIF2α	Disease model	Disease effect	References
Salubrinal	PPP1R15A(GADD34)-PP1c/PPP1R15B-PP1c	↑	ALS (SOD1G93A) Prion disease	Beneficial detrimental	[7, 88] [65]
Guanabenz	PPP1R15A(GADD34)-PP1c	↑	ALS (TDP-43) ALS (SOD1 G93A)	Beneficial	[110, 112, 118]
Sephin1	PPP1R15A(GADD34)-PP1c	↑	ALS (SOD1G93A); CMT1B	Beneficial	[21]
GSK2606414	PERK inhibitor	↓	Prion disease	Beneficial	[3, 64]
ISRIB	eIF2β	Not changed	Prion disease	Beneficial	[28, 93, 96]

*PERK* protein kinase R (PKR)-like endoplasmic reticulum kinase, *UPR* unfolded protein response, *p-eIF2α* phosphorylated eukaryotic initiation factor 2 alpha, *eIF2β* eukaryotic initiation factor 2 beta, *GADD34* growth arrest and DNA damage-inducible protein 34, *PP1c* protein phosphatase 1c, *PPP1R15A/B* protein phosphatase 1, regulatory subunit 15A/B, *ALS* amyotrophic lateral sclerosis, *CMT1B* Charcot–Marie–Tooth disease 1B, *TDP-43* TAR DNA-binding protein 43, *SOD1* superoxide dismutase 1

Sephin1, a derivative of Guanabenz without its hypotensive action, was recently demonstrated to prevent neurodegeneration in a mouse model for ALS (SOD1 G93A) as well as neuronal loss in a model for the demyelinating peripheral neuropathy Charcot–Marie–Tooth disease type 1B [21]. For treatment of a neurodegenerative process that is ongoing, however, this may be different. The synaptic loss and neurodegeneration in animal models for prion disease and Aβ pathology were attributed to chronic inhibition of translation by eIF2α phosphorylation [60, 64, 65]. Deletion of the PERK gene restores the translational defect and rescues the neurodegenerative phenotype [60, 65]. The rescue in the Aβ model is more difficult to interpret than the effects in the prion disease model, because of the direct effect of eIF2α phosphorylation on BACE1 and Aβ. In addition, the effect in the Aβ model is more related to eIF2α than PERK, because deletion of GCN2 (another eIF2α kinase) has the same effect. In the prion disease model, the interventions were initiated when pathology was already accumulating and eIF2α phosphorylation was persistent. In such a pathological state, a treatment that prolongs eIF2α phosphorylation is likely to make things worse. Indeed, in this study, decreasing eIF2α dephosphorylation by Salubrinal worsened the phenotype, whereas increasing the eIF2α dephosphorylation by overexpression of the induced phosphatase subunit PPP1R15A/GADD34 was beneficial. Another factor that may determine whether stimulation or inhibition eIF2α phosphorylation is preferred is the subcellular localization of the accumulating proteins. Reduction of synthesis of proteins that accumulate in the ER may be beneficial, whereas inhibition of synthesis of cytoplasmic proteins may only lead to further synaptic loss and neurodegeneration. With the development of GSK2606414, an ATP competitive small molecule inhibitor of the PERK kinase activity, pharmacological intervention upstream in the PERK signaling pathway became feasible [3]. Treatment with the PERK inhibitor ameliorated neurodegeneration similar to the genetic interventions in the PERK pathway [64]. This provides an interesting proof of concept for involvement of PERK, however, inhibition of PERK is associated with severe pancreas pathology, as was also observed in the PERK knockout mouse [31, 129]. More recently, ISRIB (ISR Inhibitor B) was identified, which targets the translational arrest downstream of eIF2α and thus circumvents PERK [96, 97]. It was demonstrated to act at the level of the exchange factor eIF2β and has positive effects on memory formation [93]. In the prion disease mouse model, ISRIB was indeed reported to ameliorate pathology. Although somewhat less effective than the PERK inhibitor in protection against neurodegeneration ISRIB showed strongly reduced pancreatic toxicity [28]. It is important to note that all treatments that target downstream of PERK are not UPR specific, but will affect the ISR in general.

The UPR is a homeostatic stress response. This implies that it is heavily regulated via positive and negative feedback loops. There is crosstalk between the three signaling pathways, so modulation of one pathway will affect signaling through the other two pathways as well. In a simple metaphor, this compares to the inhibition of water to flow through a tube on one end, which increases pressure elsewhere in the tube. Therefore, inhibition of one pathway may in fact increase signaling through one of the other pathways. For example, deletion of PERK results in increased activity of IRE1α [31]. The connection between the site of intervention and the effect on the neurodegenerative process is therefore not always direct. This is not necessarily negative, an example of that is demonstrated in mice deficient for XBP-1 [37]. It was expected that incapacity to activate the XBP-1 transcriptional response would worsen the phenotype of a SOD1 mouse model for fALS. In contrast, it was shown to provide protection in this neurodegenerative model. This was attributed to increased autophagic clearance of SOD1 aggregates. It is tempting to speculate that inhibition of the IRE1 pathway results in increased signaling via the PERK and ATF6 pathways as both pathways, predominantly PERK, were shown to activate autophagy [99, 116]. In a mHtt transgenic model for HD, the deletion of XBP-1 was also found to be protective and accompanied by increased autophagic clearance of the aggregates [114]. However, in this model deletion of ATF4 alone had no effect on pathology. Instead, the findings suggested the activation of the Forkhead box O1 transcription factor. In addition, the HD model mice showed activation of the IRE1 pathway only, indicating it was different from canonical UPR activation to start with. Also in the studies addressing deletion and inhibition of PERK in neurodegenerative mouse models, there was no clear evidence of canonical UPR activation [60, 64, 65]. In this respect, we should be aware of potential ER stress-independent functions of the major UPR factors as well.

## Concluding remarks and perspective

**Fig. 3:**
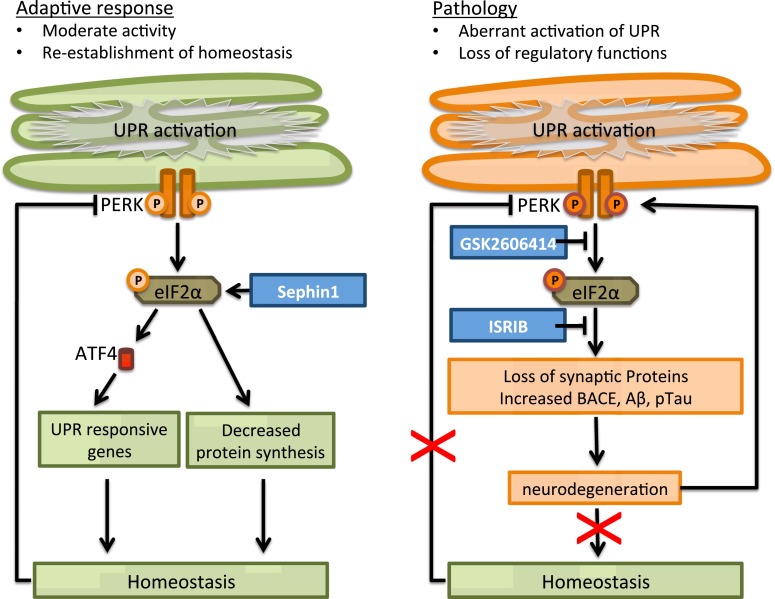
The adaptive and maladaptive PERK pathway in neurodegenerative disease. In several neurodegenerative diseases, the PERK pathway is activated. The adaptive PERK pathway (*left*) functions to restore ER proteostasis. In contrast, in pathology (*right*) prolonged activation in neurodegenerative disease leads to loss of regulatory feedback and turns the adaptive UPR maladaptive, leading to accumulation of aberrant Aβ en tau proteins and loss of synaptic proteins. It may be beneficial to stimulate the PERK pathway (e.g., by Sephin1) in the adaptive state, however, this may worsen the situation in the pathological state. Inhibition (e.g., by GSK2606414 or ISRIB) rather than stimulation of the pathway may therefore be beneficial for neurodegenerative diseases associated with persistent UPR activation. See text for further details

Evidence for UPR activation can be found in patient brains as well as models of several neurodegenerative diseases. The list of small molecules that target the UPR is growing. It is however important to distinguish positive and negative effects of the UPR. This is complicated by the notion that the direction in which to interfere (stimulation or inhibition) may be strongly affected by the pathological state. Caution is therefore warranted to directly translate mechanistic observations in the physiology to an application in pathology, where the adaptive UPR may have turned maladaptive. For example, the PERK pathway is activated in several neurodegenerative diseases, in the presence or absence of activation of the other UPR pathways. The adaptive PERK pathway functions to restore ER proteostasis by reducing overall protein synthesis via phosphorylation of eIF2α and increasing the expression of UPR responsive genes via the production of the transcription factor ATF4. PERK activation increases BACE levels and thus Aβ formation. In addition, tau phosphorylation is increased if PERK is activated. The function of these transient events in the adaptive response is not fully elucidated. During prolonged UPR activation, however, aberrant Aβ and tau proteins will accumulate which will facilitate pathology and in turn may contribute to UPR activity directly or indirectly in a vicious cycle. In addition, the persistent inhibition of protein translation results in loss of synaptic proteins that are essential for neuronal function. The prolonged UPR activation in the pathological state turns the adaptive UPR maladaptive (Fig. 3). This has important implications when using intervention in this pathway as therapeutic strategy. For example, prolonged phosphorylation of eIF2α by Guanabenz or Sephin1 may be beneficial in prevention paradigms. However, in a pathological state with persistent eIF2α phosphorylation at the start of treatment this may take a turn for the worse and inhibition of the pathway is preferred, however, ISR activators like sephin1 may be beneficial in case of accumulation of ER retained proteins.

Better understanding of the pathological state is pivotal to make a next step in UPR targeting for treatment of neurodegeneration. This will involve more precise characterization of the nature of the disturbance in the different pathways, for example, delineation of the involvement of GVD. In addition, new insights in the pathogenesis of neurodegenerative diseases like the spreading of pathological proteins will have to be incorporated into the bigger picture. These are a few of the issues that need to be addressed in the coming decade of UPR research in neurodegeneration.
